# Optimization of Thermoultrasound Process of Soursop (*Annona muricata*) Nectar and Comparison of Its Physicochemical Properties and *In Vitro* Bioaccessibility of Antioxidants with Pasteurized Sample

**DOI:** 10.17113/ftb.61.04.23.8180

**Published:** 2023-12

**Authors:** Quinatzin Yadira Zafra-Rojas, José Luis Jiménez-Hernández, Enrique Javier Olloqui, Nelly del Socorro Cruz-Cansino, Ernesto Alanís-García, Esther Ramírez-Moreno, José Alberto Ariza-Ortega, Juan Carlos Moreno-Seceña

**Affiliations:** 1 Interdisciplinary Research Center, Academic Area of Nutrition, Institute of Health Sciences, Autonomous University of the State of Hidalgo, Ex Hacienda La Concepcion Circuit S/N, Pachuca-Actopan Road, C.P. 42160. San Agustin Tlaxiaca, Hidalgo, Mexico; 2 CONACyT, Postgraduate College, Campus Montecillo, Mexico-Texcoco Road, km 36.5, Montecillo, Texcoco, Estado de Mexico, Mexico

**Keywords:** functional beverage, safety, optimization, response surface methodology, total dietary fiber, phenolic content

## Abstract

**Research background:**

Soursop nectar contains antioxidants and is preserved by pasteurization. However, this technology impairs its physicochemical properties and bioactive compounds. An alternative is therefore thermoultrasound, which could counteract these effects. The thermosonicated nectar was compared with a pasteurized one and the *in vitro* bioaccessibility of antioxidants was estimated.

**Experimental approach:**

The soursop nectar (25 %) was processed and the response surface methodology was used to determine the optimal conditions for thermoultrasound treatment (TUS). The TUS (75–90 % amplitude, 3.15–15 min) was applied, and 2 % stevia and 6 % agave inulin were added as sweeteners. The microbiological, physicochemical, enzymatic and antioxidant properties were analyzed. The properties of thermosonicated nectar obtained under optimal conditions were compared with pasteurized nectar. In addition to the above determinations, microstructure, total dietary fiber (TDF) and *in vitro* bioaccessibility of antioxidants were determined.

**Results and conclusions:**

The response variables that fit the mathematical model were L*, b*, chroma (C*), total phenolic content (TPC) and antioxidant activity determined by ABTS^•+^, DPPH˙ and Fe(III) reducing antioxidant power (FRAP). The L* and DPPH˙ were affected by quadratic time and TPC by time (p<0.0001). The optimum TUS condition was 82 % amplitude for 9.15 min and the responses variables were L*, b* and C* (45.48, 3.55 and 3.62, respectively), TPC expressed as gallic acid equivalents (38.40 mg/100 mL), ABTS^•+^ expressed as Trolox equivalents (TE) (31.28 μmol/100 mL), DPPH˙ expressed as TE (124.22 μmol/100 mL) and FRAP expressed as Fe(II) (3.06 μmol/100 mL). Compared to the pasteurized sample, thermosonicated sample had high values of L* (45.56), *h*° (-56.49), TPC (26.63 mg/100 mL), ABTS^•+^ and DPPH˙ (22.03 and 129.22 μmol/100 mL, respectively), FRAP (3.10 μmol/100 mL) and low pectin methylesterase (PME) activity (0.28 U/mL). For *in vitro* bioaccessibility, thermosonicated nectar showed high absorption of TPC (15.26/100 mL) and high antioxidant activity determined by ABTS (34.92 μmol/100 mL) and FRAP (7.88 μmol/100 mL).

**Novelty and scientific contribution:**

The thermoultrasound improves the physicochemical properties and *in vitro* bioaccessibility of antioxidants in soursop nectar. On the other hand, as an alternative, this beverage offers low-calorie alternative with prebiotic properties that benefits consumer health.

## INTRODUCTION

Soursop (*Annona muricata*) belongs to the Annonaceae family and it is cultivated in Southeast Asia, South America and tropical regions of Africa (*1*). The fruit is mainly consumed fresh, as juice or nectar. Its pulp is juicy, has a pleasant smell, mildly acidic taste and contains antioxidants such as vitamin E, vitamin C, carotenoids and phenolic compounds, which are associated with the prevention of non-communicable diseases (diabetes and hypertension) as well as antiproliferative effects of cancer cells and the induction of apoptosis (*2*).

Pasteurization is generally used to extend the shelf life of processed products such as nectar. However, this conventional technology causes the degradation of the bioactive compounds, the loss of nutrients and unfavourable changes in physical properties (stability, viscosity, colour, *etc.*) due to the application of high temperatures (*3*). Therefore, several alternatives are being explored, such as thermoultrasound (TUS), which combines the use of ultrasound with temperatures below 100 °C (usually 50 and 60 °C) (*4*). The TUS technology reduces the enzymatic activity and microbiological load, and also releases bioactive compounds from the food matrix with slight changes in nutritional properties (*5*). This technology has been applied in studies such as nectar and juice (jackfruit and beetroot) using response surface methodology as a tool in research to optimize multiple responses simultaneously, with the aim to reduce the tests and experimental time (*3*, *6*).

Furthermore, due to their importance for human health, it is necessary to estimate the absorption of bioactive compounds. This was investigated in our work with beetroot juice using the *in vitro* bioaccessibility method (*6*). This method provides information on the amount of compounds present in the intestine that are released by the digestive process (chewing, enzymes, pH) and could be absorbed through the intestinal barrier (*7*). Finally, the high consumption of simple sugars is a worldwide problem, so one of the alternatives could be the addition of natural sweeteners with low calorie content and health benefits, such as stevia (glycoside) and inulin (hydrocolloid) (*8*, *9*). These hypocaloric products have been added to different nectars (orange, pomegranate, guava and mango) (*10*, *11*). The aim of this study is to determine the optimal conditions for processing soursop nectar with thermoultrasound, taking into account its microbiological, physicochemical, enzymatic and antioxidant properties and comparing the *in vitro* bioaccessibility of its antioxidants in the intestine with a pasteurized sample.

## MATERIALS AND METHODS

### Plant material and nectar preparation

According to Codex Alimentarius, nectar is the unfermented but fermentable product obtained by adding water with or without the addition of sugar, honey and/or syrup and/or sweeteners as a food additive to a mixture of these products (*12*). For the production of soursop nectar, 10 kg of fruit at a stage of commercial maturity and without external damage were purchased at the local market in Pachuca, Hidalgo, Mexico. The fruits were washed and disinfected with a previously prepared solution of 0.082 % ionized silver (Microdyn®, Mexico City, Mexico), and then the skin and seeds of the soursop were removed. The nectar was prepared as 25 % solution by the addition of purified water according to Codex Alimentarius (*12*) and homogenization in a blender for 30 s. Immediately after the preparation of the nectar, it was treated with thermoultrasound in the first stage of the study to determine the optimal process conditions. Then, the nectar preparation was repeated to obtain a control (untreated nectar), a pasteurized nectar and the optimized thermosonicated sample for their comparison.

### Thermoultrasound and pasteurized treatments

A high intensity ultrasonic equipment (VCX-1500; Sonics Materials, Inc., Newtown, CT, USA) at 1500 W and a probe with a tip diameter of 25 mm connected to an amplitude transformer at a constant frequency of 20 kHz were used. According to previous studies (*13*, *14*), the samples were thermosonicated at different amplitudes (72, 75, 82, 90 and 93 % corresponding to acoustic energy density of 3.60, 3.75, 4.10, 4.50 and 4.65 W/mL, respectively) and times of 3.15, 5, 9.15, 13.30 and 15 min with pulse duration of 2 s on and 4 s off. A sample of 300 mL was heated in a jacketed vessel through which water circulated in a water bath (12108-10; Cole-Parmer, Vernon Hills, IL, USA) to obtain a sample outlet temperature of (50±2) °C. On the other hand, pasteurization was carried out at 65 °C for 30 min (*15*) using the same jacketed vessel and volume of the sample (300 mL). The samples (including control) were collected in sterile flasks immediately after treatment. According to Alizadeh *et al*. (*11*), 2 % of stevia as commercial sweetener and 6 % of agave inulin were added. The microbiology, colour, pH, total soluble solids, titratable acidity, viscosity and stability were measured on fresh samples, while for the rest of the determinations, the samples were frozen at -35 °C for one week.

### Optimization of thermoultrasonication process using response surface methodology

A central composite rotatable design was used, where two independent variables (amplitude and sonication time) each one at five levels (−α, −1, 0, +1, +α) were analyzed. The amplitudes of 75–90 % (X_1_) and time of 3.15–15 min (X_2_) were used. The design yielded 13 treatments, where 5 were the central points, 4 factorial points and 4 axial points (Table S1), with a distance of α=1.414 between each central design. The multiple linear regression of the dependent variable data obtained in triplicate was analyzed and the mean value of each attribute was taken as the response (Yi) using JMP® v. 7.0.2 statistical software (*16*) and fitted to a second order polynomial model, given in the following equation:



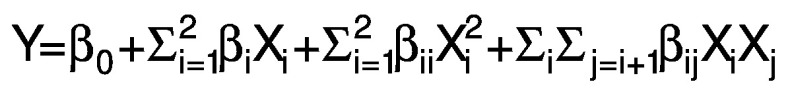



where Y and β_0_ correspond to the predicted response and the constant coefficient, respectively, β_i_ and β_ii_ are linear and quadratic coefficients, respectively, β_ij_ is the interaction coefficient, and X_1_ and X_2_ are the independent variables that correspond to the amplitude and sonication time, respectively.

To establish the optimal process conditions, the response variables that fit the mathematical model with a coefficient of determination (R^2^) and adjusted R^2^ (R^2^_adj_) both ≥0.80 were used. The regression coefficient, the significance level (p) and the predicted values were also obtained. Three-dimensional and contour figures were made using SigmaPlot v. 12.3 software (*17*). The optimal TUS conditions were visually detected by overlapping contours figures. The reproducibility of the optimal process conditions was checked in three repetitions measured in triplicate (*N*=9) and compared with the predicted values using a Student’s *t-*test with a significance level of p<0.05 (SPSS v. 15.0) (*18*).

### Microbiological analysis

Microbiological determination was done by the microdrop method. An aliquot of 100 μL of sample diluted in previously sterilized 0.1 % peptone water was prepared. Three decimal dilutions at 1:10, 1:100 and 1:1000 and a direct inoculation (20 μL) were carried out. Aerobic mesophilic microorganisms were determined using plate count agar medium and were incubated (LSI-3016A; Labtech, Gyeonggi-do, Korea) for 48 h at 30 °C. For *Enterobacteriaceae* count, the samples were poured in violet red bile glucose agar (VRBG) and incubated for 24 h at 37 °C. The colony count was expressed as log colony forming units (CFU) per mL.

### Quality measurements

The pH was measured using a potentiometer (PH210; Hanna Instruments, Nusfalau, Romania), while for total soluble solids (TSS) (expressed in °Brix) a refractometer (Brix/ATC FG-113; Hangzhou Chincan Trading Co., Ltd., Hangzhou, PR China) was employed. The titratable acidity was measured and the results were expressed in percentage.

### Physical and chemical parameters

The browning index was determined according to the method described by Martins *et al.* (*19*) with minor modifications. A volume of 10 mL of sample was centrifuged (V6500; Hamilton Bell, Montvale, NJ, USA) for 10 min at 1321×*g* to remove coarse particles. Then, 5 mL of the supernatant were taken and added to another centrifuge tube with 5 mL of 95 % ethanol (Sigma-Aldrich, Merck, Dublin, Ireland) and were centrifuged under the above conditions. Following that, 200 μL of the supernatant were aggregated in a microplate and the absorbance was measured at 420 nm with a microplate reader (Power Wave XS; BioTek Instruments Inc, Winooski, VT, USA) equipped with KCjunior software.

The viscosity was determined with DV3T rheometer (Brookfield Engineering Laboratories, Middleboro, MA, USA) using the LV-4 needle at a speed of 60 rpm. For analysis, 35 mL of sample were placed in a 50-mL centrifuge tube at 20 °C. The results were expressed in mPa∙s.

To determine physical stability, 10 mL of sample were centrifuged in a previously weighed 15-mL centrifuge tube (V6500; Hamilton Bell) at 1321×*g* for 20 min and the supernatant was removed. The stability was calculated by mass difference and expressed in percentage.

The cloud index was determined according to Cervantes-Elizarrarás *et al.* (*14*). An aliquot of 5 mL was centrifuged (V6500; Hamilton Bell) at 1321×*g* for 10 min. Then, 200 μL of the supernatant were added to a microplate and the absorbance was measured at 660 nm with a microplate reader (Power Wave XS; BioTek Instruments Inc.), taking the absorbance value as the cloud index. Distilled water was used as blank.

Pectin methylesterase (PME) enzyme was determined by titration of the carboxyl group (*20*). It was carried out by adding 40 mL of 1 % citrus pectin in a 2 mol/L NaCl solution with 10 mL of soursop nectar. The mixture was adjusted with 1 mol/L NaOH to obtain pH=7.0, then 1 mL of 0.05 mol/L NaOH was added and the time was measured until pH=7.0 was reached. The results were expressed in pectin methylesterase unit (PMEU), which is equivalent to 1 μmol of carboxyl group per minute at pH=7.0 and 30 °C, calculated according to the following equation:

PMEU=(*V*(NaOH)∙*c*(NaOH))/(*V*(sample)∙*t*) /2/

### Colour parameters

A portable colorimeter (CM-600d; Konica Minolta, Tokyo, Japan) was used to measure CIELAB parameters, where L* is lightness (0=black, 100=white), while a* and b* are red/green and blue/yellow coordinates, respectively. Saturation or chromaticity (C*) and hue (*h*°) were calculated using a* and b* values:

C*=(a*^2^ + b*^2^)^1/2^ /3/

*h*°=arctan(*b*/*a*) /4/

To compare colour differences, the values of control and pasteurized nectars were used as reference. The total colour difference or ΔE* was obtained from the L*, a* and b* values using the following equation (*21*):

ΔE* = [(ΔL*)^2^ + (Δa*)^2^ + (Δb*)^2^]^½^ /5/

### Total phenolic content, antioxidant activity analysis by ABTS^•+^, DPPH˙ and Fe(III) reducing antioxidant power

The total phenolic content (TPC) was determined using the Folin-Ciocalteu method according to Stintzing *et al*. (*22*). The solution of Folin-Ciocalteu/distilled water 1:10 was prepared and a calibration curve was prepared at *γ*(gallic acid)=0, 100, 200 and 300 mg/L. Briefly, 100 µL of the sample, 500 µL of Folin-Ciocalteu (1:10) solution and 400 µL of sodium carbonate (7.5 %) were added to an Eppendorf tube, vortexed and incubated for 30 min at room temperature. Later, the absorbance was measured at 765 nm in a microplate reader (Power Wave XS; BioTek Instruments Inc.). The results were expressed in milligrams of gallic acid equivalents (GAE) per 100 mL.

The ABTS^•+^ scavenging activity was determined according to Kuskoski *et al*. (*23*). The radical cation ABTS^•+^ was produced by reaction of two compounds (7 mmol/L ABTS and 2.45 mmol/L potassium persulfate in distilled water) in the dark at room temperature for 16 h before use. The ABTS^•+^ solution was then diluted with deionized water until reaching an absorbance of 0.70±0.10 at 754 nm. A calibration curve at concentrations of *c*(Trolox)=0, 5, 10, 20, 30, 40 and 50 µmol/L was prepared. After that, 100 µL of sample and 900 µL of diluted ABTS^•+^ were added to an Eppendorf tube and incubated at room temperature for 7 min. The absorbance of the mixture was measured at 754 nm in a microplate reader (PowerWave XS; BioTek Instruments Inc.). The results were expressed in µmol of Trolox equivalents (TE) per 100 mL.

The DPPH˙ scavenging activity was determined according to the method described by Morales and Jiménez-Pérez (*24*). The stable *γ*(DPPH radical)=7.4 mg/100 mL was prepared in ethanol. The calibration curve was prepared with standard solutions of *c*(Trolox)=0, 50, 100, 200 and 300 µmol/L. Then, 100 μL of sample and 500 μL of DPPH˙ solution were mixed in an Eppendorf tube and left to stand for 1 h at room temperature. The absorbance was measured at 520 nm using a microplate reader (Power Wave XS; BioTek Instruments Inc.). The results were expressed in µmol of TE per 100 mL.

The Fe(III) reducing antioxidant power (FRAP) was determined according to Thaipong *et al*. (*25*). The FRAP solution was prepared from a mixture of three solutions (10:1:1) as follows: 300 mmol/L acetate buffer (3.1 g C_2_H_3_NaO_2_·3H_2_O and 16 mL C_2_H_4_O_2_ (pH=3.6)), 10 mmol/L TPTZ (2,4,6-tripyridyl-*s*-triazine) solution in 40 mmol/L HCl, and final solution 20 mmol/L FeCl_3_·6H_2_O. The FRAP solution was then placed in a water bath at 37 °C. A calibration curve was made with standard solutions of *c*(Fe(II))=0, 20, 30, 40 and 50 µmol/L. Then, 30 µL of sample, 90 µL of distilled water and 900 µL of the FRAP solution were placed in an Eppendorf tube, mixed and stored in the dark for 10 min. Finally, absorbance was measured at 593 nm with a microplate reader (Power Wave XS; BioTek Instruments Inc.) and the results were expressed in µmol of Fe(II) per 100 mL.

### Comparison of parameters of optimized thermoultrasound and pasteurized soursop nectar

After the optimal conditions for TUS were obtained, the treated samples were compared with a pasteurized soursop nectar and the untreated sample (control). In addition to the parameters described above, dietary fiber content (total, soluble and insoluble), microstructure and *in vitro* intestinal bioaccessibility of antioxidants were determined.

### Dietary fiber content

A lyophilized sample (FreeZone 6; Labconco, Kansas City, MO, USA) of 5 g was milled (A11 basic; IKA, Wilmington, NC, USA) and sieved (500 μm) and then used for total dietary fiber (TDF) and soluble dietary fiber (SDF) determination. Enzymatic-gravimetric method with a commercial kit for TDF assay (TDF-100A; Sigma-Aldrich, Merck, St Louis, MO, USA) was used under the specifications given by the manufacturer following the AOAC method 985.29 (*26*). The results were expressed in percentage. The insoluble dietary fiber (IDF) was calculated by the difference between the total and soluble fiber using the following equation:

IDF=TDF-SDF /6/

### Microstructure analysis by scanning electron microscopy

To determine the microstructure of the soursop nectar, a scanning electron microscope (SEM) (JSM-IT300; JEOL, Peabody, MA, USA) with magnifications of 250 and 500 was used. A lyophilized sample was fixed on a double-sided graphite tape, coated with a 1 mm thin layer of gold and placed in a sputter coater (Denton Vacuum LLC, Moorestown, NJ, USA), where a pressure of 2.67 Pa and a current of 20 mA for 4 min was applied.

### Determination of in vitro bioaccessibility of total phenolics and antioxidant activity

*In vitro* bioaccessibility of total phenolics and antioxidant activity were determined using an *in vitro* digestion model followed by dialysis, based on the method described by Ramírez-Moreno *et al*. (*27*). A sample of 20 mL was adjusted with 6 mol/L HCl to pH=2.0, then incubated and held under continuous shaking (Allegra 25TM; Beckman Coulter, Palo Alto, CA, USA) with 120 µL of pepsin solution (40 mg pepsin (P-7000; Sigma-Aldrich, Merck) per mL 0.1 mol/L HCl) at 37 °C for 2 h. Then, 1.5-mL solution of pancreatin, sodium cholate and sodium deoxycholate (5 mg pancreatin, 12.5 mg sodium cholate hydrate and 12.5 mg sodium deoxycholate per mL of 0.1 mol/L NaHCO_3_) was added. The solution was placed in a dialysis membrane (12–14 kDa molecular mass cut-off, width 35 mm; Sigma-Aldrich, Merck) and dialyzed in 250 mL of sodium bicarbonate solution at pH=7.5 for 16 h with gentle stirring (60 rpm). Aliquots of dialyzed fraction (bioaccessible fraction) were taken and phenolic compounds and antioxidant activity (ABTS^•+^, DPPH˙ and FRAP) were determined. The bioaccessibility (in the small intestine) of TPC and antioxidant activity were calculated as a difference between the values obtained for the original sample (before *in vitro* digestion) and the sample in the dialyzed fraction.

### Statistical analysis

All determinations were done by triplicate and expressed as mean value±standard deviation (S.D.) (*N*=9). The differences among the optimized thermosonicated soursop nectar, pasteurized sample and control were analyzed with analysis of variance (ANOVA) and the Duncan’s multiple range test at level significance p<0.05, using the SPSS® 15.0 software for Windows (*18*).

## RESULTS AND DISCUSSION

### Optimization of thermoultrasonication process

As previously described, to obtain the optimal conditions of the thermoultrasound (TUS) treatment of soursop nectar, the response surface methodology was used. According to the experimental design and mathematical model, the response variables (dependents) were used to obtain a determination coefficient (R^2^) and adjusted coefficient of determination (R^2^_adj_) ≥0.80 (80 %). Therefore, in the present study the response variables that fit the mathematical model were L*, b*, C*, TPC, antioxidant activity determined with ABTS^•+^, DPPH˙ and FRAP methods (which will be described in more detail in the colour section). The response variables that did not fit the mathematical model (data not shown) were microbiology, pH, TSS, titratable acidity, browning index, viscosity, stability, cloud index and PME. However, the results obtained for these variables are described below.

### Effect of thermoultrasound on aerobic mesophilic bacteria and Enterobacteriaceae count in soursop nectar

The untreated soursop nectar (control) had values of 5.24 log CFU/mL for aerobic mesophilic and 5.00 log CFU/mL for *Enterobacteriaceae* count. All thermosonicated nectars showed a reduction of 1.42–3.54 log CFU/mL of aerobic mesophilic bacteria compared to control sample with lower microbial load of 1.70 log CFU/mL at 75 % amplitude for 13.30 min. Notably, the growth of *Enterobacteriaceae* was not observed in the samples thermosonicated at 75 % amplitude for 13.30 min, at 82 % amplitude for 15 min and at 93 % amplitude for 9.15 min (Table S1). These treatments were within the international criteria (<2 log CFU/mL) for aerobic mesophilic and *Enterobacteriaceae* in pasteurized juices (*28*), except for aerobic mesophilic count in the treatment at 93 % amplitude for 9.15 min. The reduction in bacterial load could be due to the weakening of the cell wall since thermoultrasound causes cavitation (regions with high temperatures and pressures, *i.e.* microstreaming), generating intracellular lesions, perforations and consequently exposes the cytoplasmic content (*5*).

### pH, total soluble solids, titratable acidity and browning index of thermosonicated soursop nectar

The samples (including the control) had pH values between 3.71 and 4.37. The TSS of all samples was 11 °Brix, while the titratable acidity was between 0.13 and 0.27 %. The untreated soursop nectar had browning index 0.05, while thermosonicated samples had values from 0.02 to 0.11 (Table S1). A similar behaviour was reported in another study of thermosonicated soursop nectar with 35 % pulp (*15*).

### Effect of thermoultrasound on the viscosity, physical stability, cloud index and pectin methylesterase activity of soursop nectar

The viscosity and cloud index values of the samples treated with TUS were high (309.3 to 432.7 and 0.16 to 0.25 mPa∙s, respectively) compared to the control (220.7 and 0.15 mPa∙s, respectively). Thermosonicated samples had low physical stability (44.0 to 62.7 %) compared to the untreated nectar (63.2 %). Regarding the activity of PME, the control sample had 0.34 U/mL and treated soursop nectars had 0.22 to 0.33 U/mL (Table S1). The viscosity of the treated samples increased because the ultrasound caused the fragmentation of large particles due to cavitation and temperature during sonication increased the solubility of the pectin particles (*29*). The increase of cloud index in thermosonicated soursop nectars could be due to the collapse of bubbles formed during cavitation, which disintegrates molecules and particles (*30*). The inactivation of PME is attributed to the generation of free radicals and denaturation of the enzyme during thermoultrasonication (*31*).

### L*, a*, b*, chroma, hue and total colour difference of thermosonicated soursop nectar

**Fig. 1:**
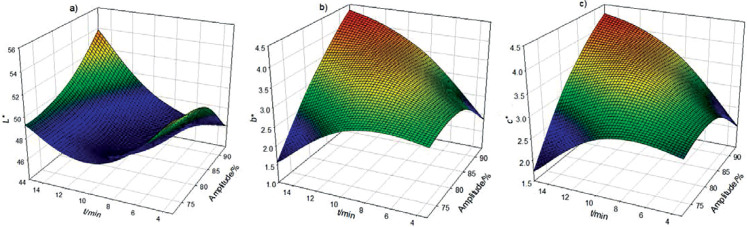
Effect of thermoultrasound on: a) L*, b) b* and c) chroma in soursop nectar

The values of L*, a*, b*, chroma (C*) and hue (*h*°) are shown in Table S2. The control nectar was slightly darker, showing low values of L* parameter (40.4) than the treated samples (44.5 to 50.7). For the a* and b* coordinates, the untreated nectar showed values of -0.57 and 3.83, respectively, while the thermosonicated samples were in range of -1.03 to -0.07 and 2.38 to 3.99, respectively, placing all samples in the green/yellow quadrant. Regarding C* and *h°*, the control soursop nectar had values 3.87 and -81.59, respectively, which were between the values found in the treated samples with TUS (2.52 to 4.01 and -88.93 to -70.75, respectively). The L*, b* and C* parameters had R^2^ of 0.94, 0.93 and 0.95 and R^2^_adj_ of 0.89, 0.88 and 0.91, respectively, showing high correlation degree. According to the regression coefficient results, β_22_ (time×time) significantly (p<0.0001) affected the L* parameter (Table 1). Fig. 1a shows that longer exposure to thermoultrasound increases luminosity. The b* coordinate was affected (p<0.001) by amplitude×time interaction (β_12_) in a way that with increasing amplitude and prolonging time, the b* value also increases (Fig. 1b). The linear term of amplitude (β_1_), the interaction term (β_12_) and β_22_ significantly (p<0.001) affected chroma (Table 1). The positive effect of β_1_ and β_12_ can be seen in Fig. 1c, where it is observable that higher amplitude and prolonged time increase the chromaticity.

**Table 1:** Model regression coefficient and significance for the response variables of thermosonicated soursop nectar

Coefficient	L*	b*	C*	TPC	ABTS	DPPH	FRAP
β_^0^_ (intercept)	45.48ª	3.55ª	3.62ª	38.40ª	31.28ª	124.22^a^	3.06ª
β_^1^_ (amplitude)	0.02	0.30^c^	0.30^b^	-0.26	4.21ª	-6.94	0.02
β_^2^_ (time)	0.18	0.15^d^	0.18^c^	4.04ª	2.94^b^	4.39	0.43^b^
β_^12^_ (amplitude×time)	1.41^c^	0.49^b^	0.43^b^	0.78	-2.00^d^	21.35^d^	0.31^c^
β_^11^_ (amplitude×amplitude)	0.90^d^	-0.21^d^	-0.21^c^	-3.53^b^	1.75^d^	-7.44	-0.20^d^
β_^22^_ (time×time)	2.46^a^	-0.30^c^	-0.28^b^	-0.51	1.22^d^	-40.25ª	-0.10
R^2^	0.94	0.93	0.95	0.95	0.95	0.91	0.91
R^2^_^adj^_	0.89	0.88	0.91	0.91	0.90	0.84	0.83

Significance levels: ^a^p<0.0001, ^b^ p<0.001, ^c^ p<0.01, ^d^ p<0.05. TPC=total phenolic content

### Effect of thermoultrasound on total phenolic content and antioxidant activity of soursop nectar

The values of TPC and antioxidant activity (ABTS^•+^, DPPH˙ and FRAP) are shown in Table S2. The thermosonicated soursop nectars had high values of TPC, expressed as GAE, and FRAP, expressed as Fe(II) (30.1 to 43.4 mg/100 mL and 2.13 to 3.59 µmol/100 mL, respectively) compared to control sample (25.90 mg/100 mL and 1.83 µmol/100 mL, respectively). The values of antioxidant activity determined by ABTS^•+^ and DPPH˙ methods (expressed as TE) of the untreated nectar were 33.36 and 90.00 µmol/100 mL, respectively, and were within the ranges obtained for the samples treated with TUS (24.56 to 41.69 and 42.89 to 141.50 µmol/100 mL, respectively).

**Fig. 2:**
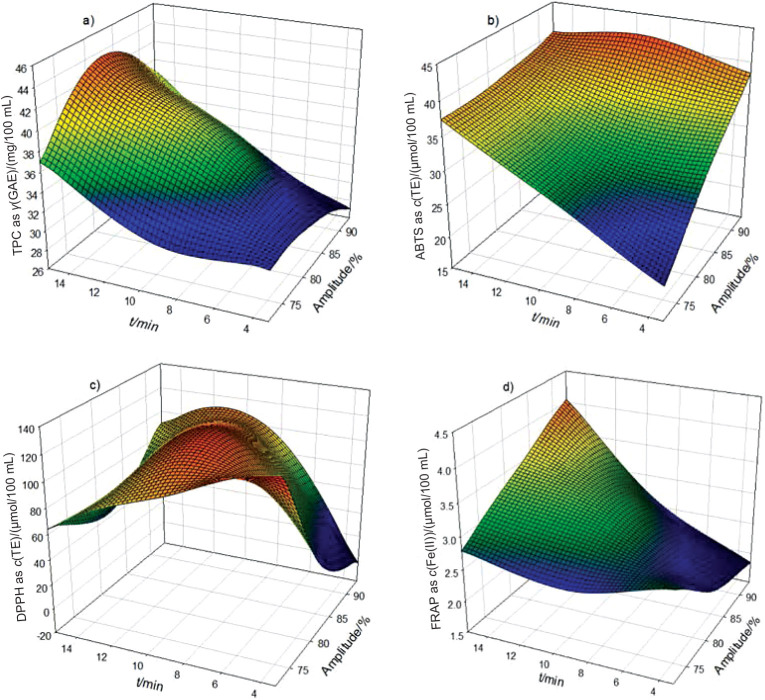
Effect of thermoultrasound on: a) total phenolic content (TPC) of soursop nectar and its antioxidant activity determined using b) ABTS, c) DPPH and d) FRAP method. GAE=gallic acid equivalent, TE=Trolox equivalent

On the other hand, all response variables showed a correlation coefficient R^2^ between 0.91 and 0.95, while R^2^_adj_ was between 0.83 and 0.91 (Table 1). According to the regression coefficient, the linear term of time (β_2_) had a significant (p<0.0001) effect on TPC (Table 1), showing an increase with prolonged thermoultrasound treatment time (Fig. 2a). Antioxidant activity determined by ABTS^•+^method was affected by the amplitude (β_1_) and time (β_2_) of thermoultrasound treatment (p<0.0001 and p<0.001, respectively) (Table 1), while Fig. 2b shows that with a higher amplitude and longer time, antioxidant activity increases. The regression coefficient *β*_22_ (time×time) had a negative effect (p<0.0001) on antioxidant activity measured by DPPH˙, while amplitude×time interaction (β_12_) had the opposite effect (p<0.05), as can be seen in Table 1. This means that increasing the time of thermoultrasound treatment caused an increase of the antioxidant activity measured by DPPH method (Fig. 2c). FRAP was significantly affected (p<0.001) by the linear term of β_2_ and interaction term β_12_ at p<0.01 (Table 1), resulting in a high antioxidant activity (Fig. 2d).

### Optimal conditions for TUS process and reproducibility of the study

As mentioned earlier, the response variables that fit the mathematical model were colour (L*, b* and C*), TPC and antioxidant activity determined by ABTS^•+^, DPPH˙ and FRAP methods. Hence, optimal conditions for the TUS were 82 % amplitude for 9.15 min and the predicted values of response variables were 45.5±0.7, 3.6±0.2, 3.6±0.1 for L*, b* and C*, respectively. TPC as GAE was (38.4±1.4) mg/100 mL, while antioxidant activity determined by ABTS^•+^ method as TE was (31.3±1.5) µmol/100 mL, DPPH˙ as TE was (124.2±14.5) µmol/100 mL and FRAP as Fe(II) was (3.06.0±0.20) µmol/100 mL.

**Fig. 3:**
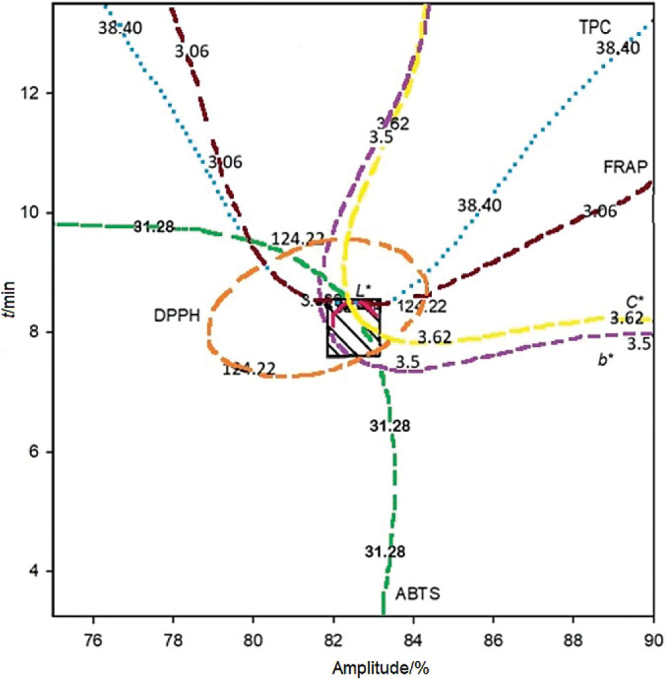
Superposition of contour plots for the response variables (L*, b*, C*, TPC as *γ*(GAE)/(mg/100 mL), ABTS as *c*(TE)/(μmol/100 mL), DPPH as *c*(TE)/(μmol/100 mL) and FRAP as *c*(Fe(II))/(μmol/100 mL)) illustrates the optimum condition (82 % amplitude for 9.15 min, marked with a square) for the thermosonication of soursop nectar. TPC=total phenolic content, GAE=gallic acid equivalent, TE=Trolox equivalent

Contour plots were made of each response variable to make an overlap between them and observe the optimal process conditions (Fig. 3). To verify the reproducibility of the optimal process conditions for thermoultrasound (82 % amplitude for 9.15 min), the experimental values were compared with the predicted values. Some colour parameters (L* and b*) and antioxidant activity (measured by DPPH˙ and FRAP) showed no significant differences (p>0.05), indicating that it is possible to reproduce the optimal TUS conditions, while C*, TPC and ABTS^•+^ had significant differences (p<0.05) (data not shown).

### Effect of treatments on aerobic mesophilic and Enterobacteriaceae count in soursop nectars

Aerobic mesophilic bacteria and *Enterobacteriaceae* were completely inactivated in the pasteurized soursop nectar and sample treated with optimized TUS, while the untreated soursop nectar had (3.65±0.06) and (3.72±0.06) log CFU/mL of aerobic mesophilic and *Enterobacteriaceae* counts, respectively (data not shown). Similar results were observed in a soursop nectar thermosonicated at 87.5 % amplitude for 10 min (*32*). Center for Food Safety (*33*) determines as satisfactory the foods with values of aerobic mesophilic and *Enterobacteriaceae* counts lower than 5.7 and 3.7 log CFU/mL, respectively. Therefore, the treated nectars complied with the established limits. The inactivation of bacteria in pasteurized soursop nectar is due to the fact that heat treatment alters the cell membrane stability, reducing the thickness of the phospholipid bilayer and as a consequence increases its permeability (*34*). In the thermosonicated sample, the damage is caused by the shearing and cavitation when pressure changes occur (compression and decompression of gas bubbles) and high temperatures, causing cell lysis (*5*).

### Effect of treatments on colour parameters of soursop nectar

The L*, a* and b* coordinates, C* and *h*°, as well as total colour difference (Δ*E*) are shown in Table 2. The soursop nectar treated with optimized TUS had a high value (p<0.05) of L* (45.56) compared to the other samples. Similar behaviour was reported in a thermosonicated soursop nectar (*32*). The increase of L* in the TUS nectar is attributed to partial precipitation of suspended particles that are not stable (*34*). The control sample had significantly (p<0.05) high values of a*, b* and C* compared to treated samples. Different results were obtained for a* and b* in the thermosonicated soursop nectar (*35*). Regarding the *h*° parameter, the optimized TUS treatment showed higher value (p<0.05) than the pasteurized and untreated nectar. Different trends in other studies are probably due to the equipment used, the origin of the fruit and the nectar formulation. With the control sample as reference, total colour difference (ΔE*) of the pasteurized soursop nectar was low compared to the optimized thermoultrasound treatment. Taking the pasteurized sample as reference, ΔE* value of the optimized TUS nectar was slightly higher than of the untreated nectar (Table 2). If Δ*E* is >3.5, the colour difference is perceivable by the human eye (*36*), so the difference between the optimized TUS sample and the pasteurized sample was not distinguishable.

**Table 2:** Colour parameters of soursop nectar samples

Coordinate	Control	Optimized TUS	Pasteurized
L*	(40.4±0.4)^c^	(45.6±0.3)^a^	(42.6±0.3)^b^
a*	(-1.24±0.06)^a^	(-1.58±0.04)^c^	(-1.39±0.06)^b^
b*	(5.3±0.2)^a^	(3.7±0.4)^c^	(4.25±0.02)^b^
C*	(5.3±0.2)^a^	(3.4±0.2)^c^	(4.47±0.02)^b^
*h°*	(-75.6±0.7)^c^	(-56.5±0.9)^a^	(-71.5±0.3)^b^
_Δ_ _E*__control_	–	5.4±0.3	2.4±0.3
_Δ_ _E*__pasteurized_	2.4±0.4	3.1±0.3	–

Results are presents as mean value±standard deviation, *N*=9. Different letters in superscript indicate significant difference (p<0.05) between the rows. Optimized TUS=soursop nectar treated at 82 % of amplitude for 9.15 min, pasteurized=soursop nectar treated at 65 °C for 30 min, TUS=thermoultrasound. L*=lightness, a*=red/green, b*=blue/yellow, C*=chroma, *h*°=hue, ΔE*_control_=total colour difference in control, ΔE*_pasteurized_=colour difference in pasteurized nectar. –indicates that control and pasteurized nectars were used as reference

### Viscosity, physical stability and pectin methylesterase activity in treated soursop nectars

The pasteurized soursop nectar had significantly (p<0.05) high viscosity, followed by the thermosonicated sample (Table 3). An opposite behaviour was reported in a thermosonicated carrot juice with orange pulp (*37*). The increase in viscosity of pasteurized nectar, possibly due to the effect of temperature on pectin and other solids in the medium, caused an increase in the degree of solubility (*38*). The high values of viscosity of the thermosonicated soursop nectar compared to the control may be due to the particle size reduction caused by the disruptive effect of ultrasound, where interactions between small particles result in an increase of viscosity (*29*).

**Table 3:** Viscosity, stability and dietary fiber content in soursop nectars

Sample	Viscosity/(mPa∙s)	Stability/%	TDF/%	SDF/%	IDF/%
Control	(151.6±11.6)^c^	(55.1±2.9)^b^	(3.58±0.01)^a^	(3.27±0.01)^b^	(0.31±0.03)^a^
Optimized thermosonicated (82 % amplitude for 9.15 min)	(186.9±10.5)^b^	(54.7±1.4)^b^	(3.53±0.05)^a^	(3.32±0.02)^a^	(0.22±0.06)^b^
Pasteurized (65 °C for 30 min)	(274.4±32.8)^a^	(67.4±1.2)^a^	(3.12±0.02)^b^	(2.94±0.03)^c^	(0.19±0.02)^b^

Results are presented as mean value±standard deviation, *N*=9. Different letters in superscript indicate significant difference (p<0.05) between the rows. TDF=total dietary fiber, SDF=soluble dietary fiber, IDF=insoluble dietary fiber

Regarding physical stability, the untreated nectar and the thermosonicated soursop nectar showed similar values (p>0.05), while the pasteurized sample had high stability (p<0.05) (Table 3). Similar behaviour was reported in a thermosonicated prickly pear juice (*13*). According to the pectin methylesterase activity values obtained in the control soursop nectar (0.40 U/mL), the pasteurized sample and the thermosonicated nectar reduced (p<0.05) the PME enzyme at (0.20±0.02) and (0.28±0.02) U/mL, corresponding to 50 and 69 % residual activity, respectively (data not shown). In thermosonicated jackfruit nectar a similar behaviour was observed (*3*). The enzyme inactivation in pasteurized soursop nectar is due to heat that causes hydrogen bond breakage, unfolding protein structures and deamination of enzymes (*39*), while thermoultrasound provokes enzyme denaturation by interaction with free radicals and shearing caused by cavitation (*31*).

### Total, soluble and insoluble dietary fiber content in treated soursop nectars

Table 3 shows the data obtained for total dietary fiber (TDF), soluble dietary fiber (SDF) and insoluble dietary fiber (IDF) contents. Control soursop nectar and TUS sample had a significantly (p<0.05) high content of TDF compared to the pasteurized nectar. The percentage of SDF in the thermosonicated nectar was significantly (p<0.05) high compared to the other samples, while the percentage of IDF was significantly high in the untreated nectar. The values obtained in the control, TUS and pasteurized samples for TDF and SDF were higher than described by Anaya-Esparza *et al*. (*15*). It has been reported that the addition of inulin increases the soluble fiber content (*40*). The increase of SDF in the thermosonicated soursop nectar may be due to joint activity of acoustic and thermal energy, and breakdown of plant membranes, which increases solubility of fibrous structures (*41*).

### Effect of treatments on the microstructure of soursop nectar

**Fig. 4:**
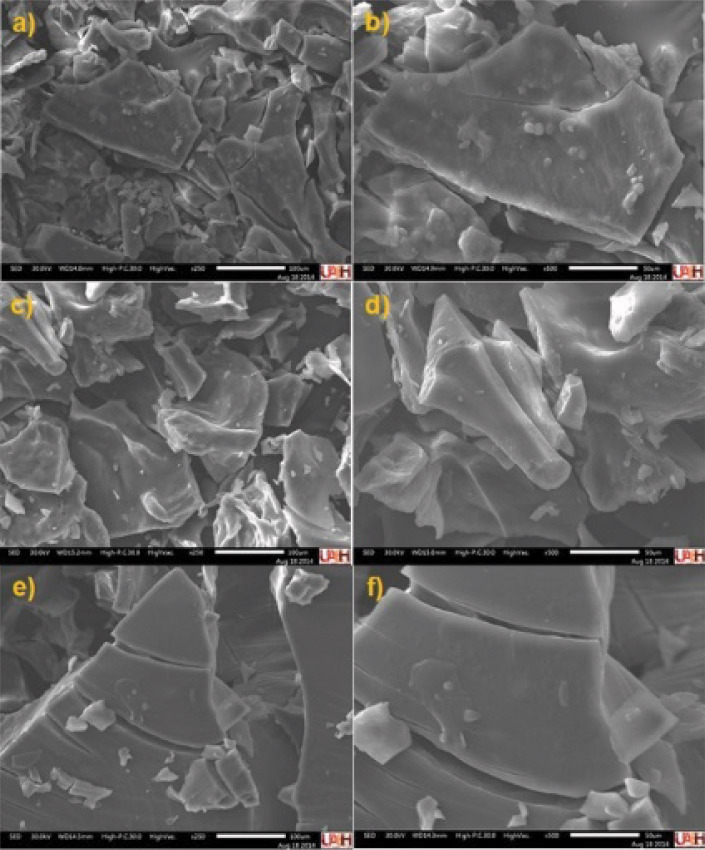
Scanning electron microscopy (SEM) of freeze-dried soursop nectar: a) and b) control at 250 and 500×, respectively, c) and d) optimized thermosonicated (at 82 % amplitude for 9.15 min) samples at 250 and 500×, respectively, and e) and f) pasteurized nectar (treated at 65 °C for 30 min) at 250 and 500×, respectively

Images obtained of soursop nectars with scanning electron microscopy (250× and 500× magnifications) are shown in Fig. 4. Amorphous structures, possibly cellulose, starch and fiber in all the samples can be observed. The control sample (Fig. 4a and Fig. 4b) and pasteurized soursop nectar (Fig. 4e and Fig. 4f) showed flake-like lamella structures with smooth surfaces, while the thermosonicated sample (Fig. 4c and Fig. 4d) showed greater fragmentation of the structures and a rough shape, which probably allowed the release of bioactive compounds from the food matrix. This was confirmed with the increase of the phenolic content and antioxidant activity (see the following section).

### Effect of treatments on total phenolic content, antioxidant activity and in vitro bioaccessibility of soursop nectar

**Fig. 5:**
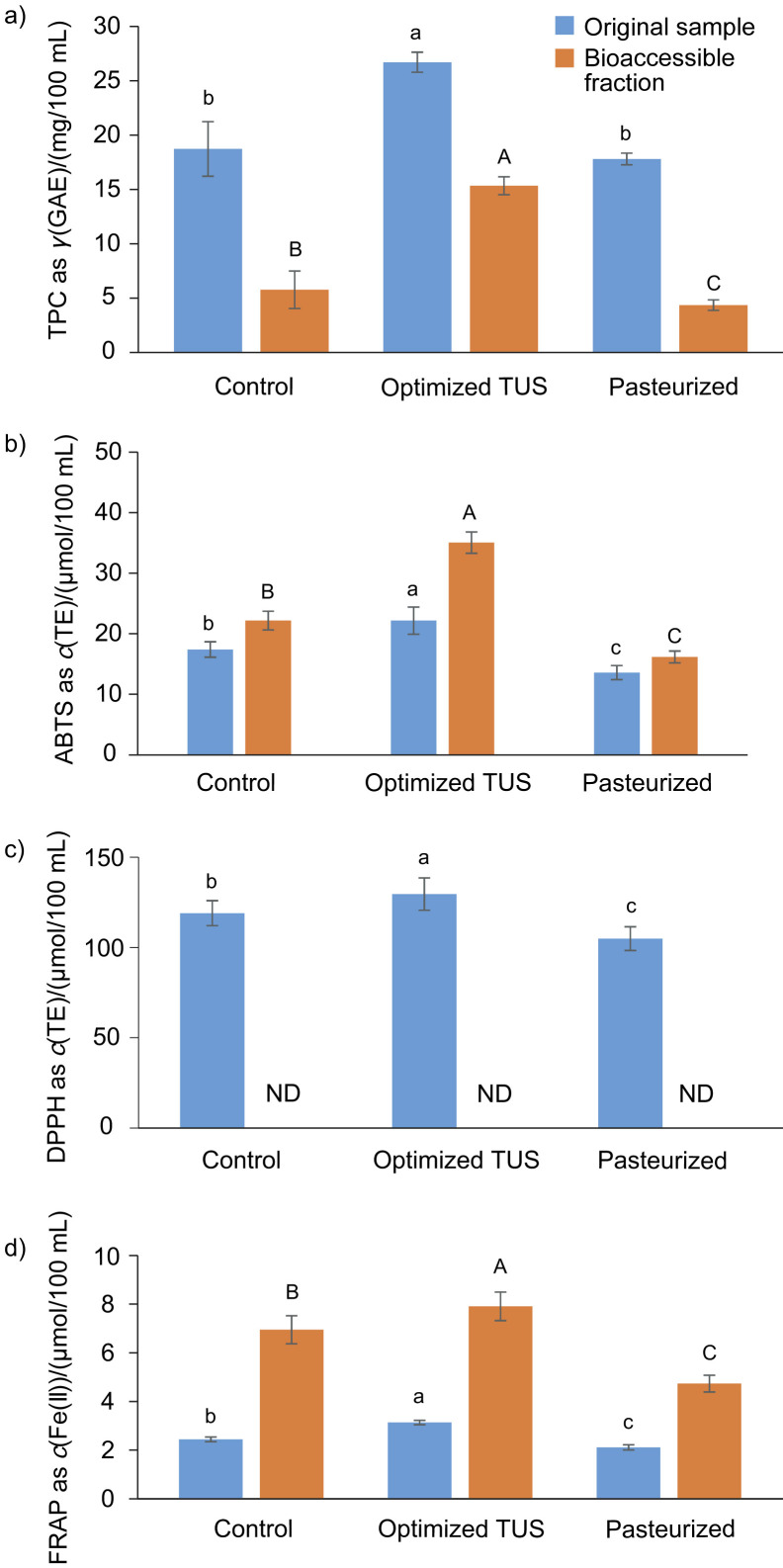
Total phenolic content (TPC) and antioxidant activity in the original sample and bioaccessible fraction of control, optimized thermosonicated (TUS) (82 % amplitude for 9.15 min) and pasteurized (65 °C for 30 min) soursop nectar: a) TPC, b) ABTS, c) DPPH and d) FRAP method. Different small letters in superscript indicate significant differences (p<0.05) between samples and different capital letters in superscript indicate significant differences (p<0.05) between bioaccessible fractions. ND=not detected, GAE=gallic acid equivalent, TE=Trolox equivalent

Fig. 5 shows the antioxidants before (original sample) and after the digestion process (bioaccessible or dialyzed fraction). In the original sample, the thermosonicated nectar had the highest value (p<0.05) of TPC content ((26.6±0.9) mg/100 mL), while the content of untreated nectar and pasteurized sample was similar (p>0.05) ((18.6±2.5) and (17.7±0.5) mg/100 mL, respectively) (Fig. 5a). A different result was reported in thermosonicated soursop nectar by Anaya *et al*. (*32*). The high content of TPC in the thermosonicated nectar may be due to the cell wall rupture, releasing the phenolic compounds in the medium (*42*).

The results for *in vitro* intestinal bioaccessibility (bioaccessible fraction) show partial bioaccessibility in all samples. The thermosonicated nectar ((15.3±0.8) mg/100 mL) showed higher value (p<0.05) than the control and pasteurized samples (Fig. 5a), with an absorption of 57.29 % compared to its original sample. A similar behaviour was found in a thermosonicated tomato juice (*43*). Partial absorption is related to the quantity of polyphenols that are released by the digestion process and solubilized in the intestinal fluid (*44*).

The antioxidant activity results of the three methods are shown in Fig. 5. The results obtained by ABTS^•+^, DPPH˙ and FRAP methods for the original sample (before *in vitro* intestinal bioaccessibility analysis) were significantly (p<0.05) higher in the TUS soursop nectar ((22.0±2.2), (129.2±9.0) and (3.10±0.09) µmol/100 mL, respectively) than in control sample ((17.3±1.3), (118.6±6.9) and (2.4±0.1) µmol/100 mL, respectively) and pasteurized nectar ((13.4±1.2), (104.5±6.6) and (2.1±0.1) µmol/100 mL, respectively) (Fig. 5b, Fig. 5c and Fig. 5d). Different results were found in the thermosonicated soursop nectar (*35*). The higher antiradical activity for ABTS^•+^ and DPPH˙ in the thermosonicated samples is attributed to the effect of sonication which generates hydroxylation in the *ortho* or *para* positions of aromatic ring of phenolic compounds, increasing the antioxidant activity (*45*) and it has been reported that phenolic acids found in soursop correlate with antioxidant activity determined by FRAP method (*46*).

After digestion (*in vitro* intestinal bioaccessibility), the TUS nectar had high (p<0.05) antioxidant activity as determined by ABTS^•+^ method ((34.9±1.8) μmol/100 mL), compared to the other samples (Fig. 5b). In all nectars, the percentage of antioxidant activity in the dialyzed fraction was more than 100 % (compared to the original samples). Similar behaviour was described in a thermosonicated beetroot juice (*6*). Regarding DPPH˙ method, antioxidant activity was not found in the nectars (Fig. 5c). Finally, the result obtained by FRAP method for thermosonicated sample was significantly high ((7.9±0.6) µmol/100 mL) compared to the other samples (Fig. 5d). All treatments showed more than double values of the antioxidant activity (FRAP) in their dialyzed fraction than in the original sample. The high antioxidant activity obtained by ABTS^•+^ and FRAP methods could be due to deprotonation of hydroxyl groups after passing from an acidic to an alkaline environment, increasing hydrogen donation (*46*). However, it can also depend on the antioxidant selectivity of each method, *e.g*. the ABTS^•+^ method is more reliable due to its solubility in both aqueous and organic solvents, in addition to a faster reaction with lipophilic and hydrophilic antioxidants than DPPH˙ (*27*).

## CONCLUSIONS

The results show that the thermoultrasound applied to soursop nectar reduced aerobic mesophilic bacteria (complying with European Commission regulations), and even totally inactivated *Enterobacteriaceae* in some thermoultrasonication treatments (75 % amplitude for 13.30 min and 93 % amplitude for 9.15 min). Physicochemical parameters in almost all treated samples remained unchanged, or only slight increase was observed in some cases. The response surface methodology is an adequate tool for the optimization of thermoultrasound treatment of soursop nectar to determine colour (L*, b*, C*), total phenolic content and antioxidant activity (ABTS^•+^, DPPH˙ and FRAP). High correlation degree, adequate fit of the mathematical model and optimal conditions of thermoultrasound processing of 82 % amplitude for 9.15 min were obtained.

The samples that were treated under optimal conditions of thermoultrasound and pasteurization had similar results in terms of microbiological quality (total microorganism inactivation), reduction of pectin methylesterase and colour (ΔE*). Both methods complied with international requirements for aerobic mesophilic bacteria and *Enterobacteriaceae*. The thermosonicated sample showed clear changes in its microstructure and high content of antioxidant compounds before (original sample) and after *in vitro* intestinal bioaccessibility (dialyzed fraction). Therefore, the soursop nectar treated with thermoultrasound can be considered a drink with greater effects on the consumer health. Further research is necessary such as product acceptability, shelf life, identification of bioactive compounds and impact on the health.

## Appendix

**Table S1:** Experimental design for determining the effect of thermoultrasound treatment on microbiological and physicochemical parameters of soursop nectar

						Response variable								
Treatment	Pattern	Amplitude/%	*t*/min	AM/(log CFU/mL)	EB/(log CFU/mL)		pH	TSS/°Brix	Titratable acidity/%	BI	Viscosity/(mPa∙s)	Physical stability/%	Cloud index	PME/(U/mL)
Control				5.2±0.3	5.0±0.4		3.91±0.03	11±0.00	0.25±0.04	0.05±0.00	220.7±7.0	63.2±1.1	0.15±0.00	0.34±0.02
1	+-	90	5.00	3.78±0.02	3.74±0.05		3.85±0.03	11±0.00	0.27±0.00	0.06±0.00	341.3±9.0	60.2±1.8	0.22±0.01	0.29±0.02
2	a0	72	9.15	3.6±0.2	3.60±0.05		3.87±0.01	11±0.00	0.22±0.04	0.08±0.00	309.3±4.2	60.0±0.5	0.21±0.00	0.27±0.02
3	0a	82	3.15	3.82±0.02	3.88±0.01		3.79±0.02	11±0.00	0.27±0.00	0.06±0.00	350.7±8.1	62.7±1.1	0.21±0.00	0.33±0.00
4^¥^	00	82	9.15	1.8±0.2	0.00±0.00		4.06±0.01	11±0.00	0.22±0.04	0.06±0.00	408±24	59.7±1.1	0.25±0.00	0.28±0.02
5^¥^	00	82	9.15	3.57±0.02	3.38±0.05		3.78±0.01	11±0.00	0.13±0.00	0.05±0.00	329±31	61.8±0.2	0.23±0.01	0.29±0.02
6	0A	82	15.00	1.8±0.2	0.00±0.00		4.04±0.02	11±0.00	0.20±0.00	0.06±0.00	381.00±10.26	59.3±0.4	0.22±0.00	0.22±0.01
7	++	90	13.30	2.8±0.2	2.65±0.05		3.71±0.01	11±0.00	0.16±0.04	0.06±0.00	364±11	56.7±0.9	0.23±0.03	0.29±0.02
8	--	75	5.00	2.2±0.2	1.8±0.6		3.75±0.01	11±0.00	0.20±0.00	0.02±0.00	433±19	53.7±1.4	0.19±0.00	0.28±0.02
9^¥^	00	82	9.15	2.6±0.1	1.70±0.00		3.72±0.01	11±0.00	0.16±0.04	0.11±0.01	398±35	56.4±0.5	0.20±0.02	0.22±0.02
10	A0	93	9.15	2.4±0.1	0.00±0.00		4.37±0.01	11±0.00	0.18±0.04	0.07±0.01	325±18	60.4±1.1	0.22±0.00	0.23±0.01
11^¥^	00	82	9.15	1.8±0.2	0.00±0.00		4.36±0.01	11±0.00	0.18±0.04	0.03±0.00	342.7±9.9	55.5±2.6	0.25±0.01	0.24±0.01
12^¥^	00	82	9.15	1.9±0.3	0.00±0.00		4.31±0.01	11±0.00	0.16±0.04	0.04±0.00	365.3±9.9	56.5±0.5	0.23±0.01	0.27±0.02
13	-+	75	13.30	1.70±0.00	0.00±0.00		4.27±0.01	11±0.00	0.18±0.04	0.03±0.00	381±33	44.0±6.4	0.16±0.01	0.25±0.01

Results are presented as mean value±standard deviation (*N*=3). ^¥^Central points. AM=aerobic mesophilic bacteria, EB=*Enterobacteriaceae*, TSS=total soluble solids, BI=browning index, PME=pectin methylesterase activity

**Table S2:** Experimental design of thermosonicated soursop nectar on colour, total phenolic content (TPC) and antioxidant activity

Treatment			Response variable								
Amplitude/%	*t*/min	*L**	*a**	*b**	*C**	*h*°	TPC as *γ*(GAE)/(mg/100 mL)	ABTS as *c*(TE)/(µmol/100 mL)	DPPH as *c*(TE)/(µmol/100 mL)	FRAP as *c*(Fe(II))/(µmol/100 mL)
Control			40.4±0.5	-0.57±0.01	3.83±0.04	3.87±0.04	-81.59±0.07	25.9±0.1	33.4±0.3	90.0±0.8	1.83±0.03
1	90	5.00	46.7±0.3	-0.78±0.04	2.63±0.06	2.75±0.06	-73.4±0.5	30.1±1.1	36.4±1.8	42.9±2.2	2.19±0.03
2	72	9.15	47.1±0.1	-0.53±0.03	2.79±0.07	2.84±0.06	-79.2±0.7	31.2±3.6	29.0±0.2	103.0±5.0	2.54±0.03
3	82	3.15	50.7±0.2	-0.38±0.02	2.86±0.04	2.89±0.03	-82.4±0.5	30.5±0.5	30.5±2.3	31.4±1.2	2.13±0.00
4^¥^	82	9.15	44.5±0.1	-0.99±0.00	3.32±0.33	3.5±0.3	-73.3±1.6	39.3±1.7	32.3±0.4	121.2±6.1	3.10±0.02
5^¥^	82	9.15	45.3±0.2	-0.75±0.02	3.4±0.3	3.4±0.3	-77.4±1.2	39.5±1.4	30.4±0.1	127.0±6.1	3.12±0.06
6	82	15.00	50.21±0.08	-1.03±0.01	3.2±0.2	3.3±0.1	-71.9±0.8	43.4±1.9	38.1±0.7	43.8±1.9	3.43±0.07
7	90	13.30	50.6±0.2	-0.33±0.02	4.0±0.2	4.0±0.2	-85.3±0.3	38.7±1.7	38.8±0.8	94.4±9.3	3.59±0.06
8	75	5.00	49.84±0.07	-0.22±0.01	2.97±0.03	2.98±0.03	-85.8±0.2	32.3±2.2	24.6±2.0	113.6±2.4	2.74±0.02
9^¥^	82	9.15	45.7±0.6	-0.07±0.01	3.8±0.1	3.8±0.1	-88.9±0.2	38.0±0.3	31.9±0.4	141.5±0.8	3.30±0.05
10	93	9.15	47.6±0.2	-0.66±0.02	3.60±0.04	3.66±0.04	-79.6±0.2	30.7±1.9	41.7±2.3	103.4±5.0	2.6±0.1
11^¥^	82	9.15	46.5±0.1	-0.54±0.01	3.63±0.03	3.67±0.03	-81.5±0.1	36.0±0.8	28.8±1.2	124.7±6.2	2.8±0.1
12^¥^	82	9.15	45.5±1.4	-0.73±0.02	3.7±0.2	3.8±0.2	-78.8±0.3	39.2±1.4	32.9±0.4	106.7±8.7	3.05±0.04
13	75	13.30	48.20±0.20	-0.83±0.04	2.4±0.1	2.5±0.1	-70.8±1.2	37.8±4.2	34.9±1.8	79.7±1.7	2.92±0.04

Results are presented as mean value±standard deviation (*N*=3). ^¥^Central points. *C**=chroma, *L**=lightness, *a**=red/green, *b**=blue/yellow, *h°=*hue, GAE=gallic acid equivalents, TE=Trolox equivalents
